# Barriers and facilitators adolescent females living with HIV face in accessing contraceptive services: a qualitative assessment of providers’ perceptions in western Kenya

**DOI:** 10.7448/IAS.18.1.20123

**Published:** 2015-09-18

**Authors:** Jill M Hagey, Eliud Akama, James Ayieko, Elizabeth A Bukusi, Craig R Cohen, Rena C Patel

**Affiliations:** 1 Medical Student, School of Medicine, University of California San Francisco, San Francisco, CA, USA; 2 Family AIDS Care & Education Services, Kisumu, Kenya; 3 Center for Microbiology Research, Kenya Medical Research Institute, Nairobi, Kenya; 4 Department of Obstetrics, Gynecology & Reproductive Sciences, University of California San Francisco, San Francisco, CA, USA;; 5 Division of Infectious Diseases, Department of Medicine, University of California San Francisco, San Francisco, CA, USA

**Keywords:** HIV, adolescents, contraception, Kenya

## Abstract

**Introduction:**

Avoiding unintended pregnancies is important for the health of adolescents living with HIV and has the additional benefit of preventing potential vertical HIV transmission. Health facility providers represent an untapped resource in understanding the barriers and facilitators adolescents living with HIV face when accessing contraception. By understanding these barriers and facilitators to contraceptive use among adolescent females living with HIV, this study aimed to understand how best to promote contraception within this marginalized population.

**Methods:**

We conducted structured in-depth interviews with 40 providers at 21 Family AIDS Care & Education Services - supported clinics in Homabay, Kisumu and Migori counties in western Kenya from July to August 2014. Our interview guide explored the providers’ perspectives on contraceptive service provision to adolescent females living with HIV with the following specific domains: contraception screening and counselling, service provision, commodity security and clinic structure. Transcripts from the interviews were analyzed using inductive content analysis.

**Results:**

According to providers, interpersonal factors dominated the barriers adolescent females living with HIV face in accessing contraception. Providers felt that adolescent females fear disclosing their sexual activity to parents, peers and providers, because of repercussions of perceived promiscuity. Furthermore, providers mentioned that adolescents find seeking contraceptive services without a male partner challenging, because some providers and community members view adolescents unaccompanied by their partners as not being serious about their relationships or having multiple concurrent relationships. On the other hand, providers noted that institutional factors best facilitated contraception for these adolescents. Integration of contraception and HIV care allows easier access to contraceptives by removing the stigma of coming to a clinic solely for contraceptive services. Youth-friendly services, including serving youth on days separate from adults, also create a more comfortable setting for adolescents seeking contraceptive services.

**Conclusions:**

Providers at these facilities identified attitudes of equating seeking contraceptive services with promiscuity by parents, peers and providers as barriers preventing adolescent females living with HIV from accessing contraceptive services. Health facilities should provide services for adolescent females in a youth-friendly manner and integrate HIV and contraceptive services.

## Introduction

In sub-Saharan Africa, 1.7 million adolescents aged 10–19 are living with HIV, with females disproportionately affected [1,2]. Large-scale changes such as the declining age of menarche, rising age of marriage and changing norms of sexual behaviour have led to earlier and more frequent sexual activity among this age group [3]. Younger age of sexual debut among adolescent females in developing countries has put them at greater risk for not only acquiring HIV, but also having unintended pregnancies [4–6]. In Kenya, only 5% of adolescents aged 15–19 use modern contraception, and an additional 29.7% have an unmet need for contraceptive services [7]. In addition, unmarried adolescents are less likely to use modern hormonal contraceptives than their older female counterparts [4]. This has led to a high fertility rate among adolescents in Kenya, ranging from 2% at age 15 to 36% at age 19 [8].

To prevent unintended pregnancy among adolescent females living with HIV, contraceptive services must reach a larger proportion of those with unmet need. Indeed, decreasing pregnancy among adolescents is associated with lower maternal morbidity and mortality; maternal mortality among adolescents is twice as high as older females [9]. Among adolescents living with HIV, avoiding unintended pregnancy reduces vertical transmission of HIV and maternal mortality associated with HIV infection [10]. Increasing contraception use among all adolescent females has also been associated with greater female empowerment and enhanced educational and economic opportunities, and adolescent females living with HIV using contraception may garner similar benefits [8,11,12].

To date, little research has been conducted on the barriers and facilitators to contraceptive services, particularly among adolescents living with HIV in resource-limited settings. Though much more is known about the barriers or facilitators that adolescents themselves identify, very few studies have examined providers’ perspectives on this topic [6,13–18]. Providers offer an important perspective, as they provide the contraceptive services adolescents need, and frequently influence community norms that dictate the acceptability of using contraception [19]. Furthermore, providers may be more aware of interpersonal, institutional and societal factors influencing contraception uptake than adolescents themselves. Health providers perceive interventions delivered at facilities as having potential to improve contraceptive uptake among female adolescents living with HIV, as these adolescents already have a reason to engage with the health facilities for their HIV care.

Research often focuses on individual factors associated with promoting a healthy behaviour without understanding the fuller context in which health behaviour decisions are made. Thus, to determine how best to promote contraceptive use among adolescent females living with HIV, we conducted in-depth interviews with HIV-care providers in western Kenya regarding contraception use among adolescents living with HIV. We created an ecologic model based on the barriers and facilitators identified across individual, interpersonal, institutional and societal levels, with each level acting as a potential target for future interventions [20,21].

## Methods

We administered a facility-level questionnaire between July and August 2014 to assess contraceptive service provision at 21 facilities supported by Family AIDS Care & Education Services (FACES), a collaboration between the University of California, San Francisco and the Kenya Medical Research Institute [22]. These facilities ranged from county and sub-county hospitals to dispensaries (Supplementary file 1 [23–25]). Facilities were located in Homabay, Kisumu and Migori counties in western Kenya with 27.1, 18.7 and 13.4% prevalence of HIV, respectively, the highest prevalence in Kenya [26]. Providers at these facilities provide comprehensive HIV and primary healthcare services, including contraception.

The facility-level questionnaire was administered to a convenience sample of one to three health providers at each facility. The lead investigator, JMH, along with Kenyan FACES staff members, visited each facility during clinic hours, and the nurses in-charge introduced us to the HIV providers involved in contraceptive service provision at their facilities. Approximately, half of these providers further specialized in antenatal care and maternal health services. All of the health providers we approached agreed and provided written consent to participate in the study. The facility-level questionnaire included open- and closed-ended questions on provision of contraception education, counselling, commodities, referrals, provider training and clinic structure (Supplementary file 2). RCP and JMH led the questionnaire development with input from all co-authors. JMH administered the questionnaire and took field notes during the interviews. In addition, 77% of providers provided written consent to be audio recorded during the open-ended questions. All health providers were compensated for their time, equivalent to approximately US$8 (680 KSh).

Content analysis was used to identify themes regarding barriers and facilitators for adolescent females living with HIV in obtaining contraceptive services. JMH and RCP independently conducted the initial coding of a sample of transcripts, and discrepancies in coding were resolved through discussion. Inductive codes were further developed as concepts emerged. Finally, codes were grouped to identify thematic trends and variant views. JMH and RCP with guidance from EA, JA and EAB organized these themes within an ecologic model [27,28]. Study data were transcribed using REDCap version 6.0, and all qualitative analyses were completed using NVivo version 10.1.1 [29,30].

The study was approved by the UCSF Committee on Human Research (CHR #13-12304) and the KEMRI Ethics Review Committee (SSC #2770).

## Results

Most of the facility providers we interviewed were female (82%) and either nursing (54%) or clinical officers (41%, Table 1). The providers’ primary work stations included county and sub-county hospitals (46%), health centres (36%) and dispensaries (18%).

**Table 1:** Characteristics of health providers interviewed at 21 FACES-supported HIV facilities

Characteristics	Number
Type of health facility	
County hospital	3 (8%)
Sub-county hospital	15 (38%)
Health centre	14 (36%)
Dispensary	7 (18%)
Types of health providers interviewed	
Clinical Officer (CO)	16 (41%)
Nursing Officer (NO)	21 (54%)
Pharmacist	1 (2%)
Community Clinic Health Assistant (CCHA)	1 (2%)
Sex of health providers interviewed	
Female	32 (82%)
Male	7 (18%)
Number of health providers interviewed per interview	
1	38 (98%)
2	1 (2%)
Location of facility	
Homabay County	10 (26%)
Kisumu County	9 (23%)
Migori County	20 (51%)

Percentages may not add up to 100% due to rounding.

**Figure 1:**
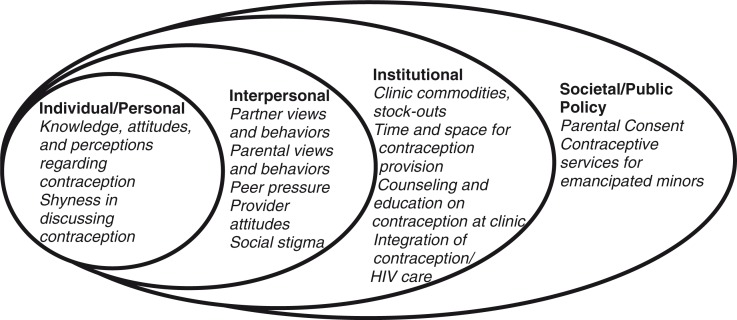
Ecologic model of barriers and facilitators for contraception use among adolescent females living with HIV.

According to the providers, several factors influenced the ability of adolescent females living with HIV to access contraceptive services from HIV-care facilities (Figure 1 and Table 2). The major barriers providers identified were interpersonal factors, such as interactions with partners, parents and peers, which prevented many adolescent females from accessing services because of the stigma of being sexually active while unmarried. On the other hand, providers identified the most important facilitators as institutional factors, such as youth-friendly services and integration of contraception into other services such as HIV and prenatal care, which promoted access to contraceptive services.

**Table 2:** Barriers and facilitators for contraception use among adolescent females living with HIV

Characteristics	Barriers	Facilitators
Individual/personal	Lack of knowledge of contraceptive methods and services Refusal of services because not ready to initiate contraception	Knowledge of contraceptive methods and services
Interpersonal	Fear of being judged for seeking services without male partner Community stigma for being sexually active and seeking services Fear of parental attitudes in seeking services Provider biases in not providing hormonal contraception methods Fear of stigma by health providers preventing contraception discussion	Peer encouragement to seek services Positive staff attitudes Provider confidentiality in providing services
Institutional	Health facility barriers, such as physical environment, scheduling and language differences among providers and adolescents	Youth-friendly service provision Integration of HIV and contraception services for ease of accessing care Appropriate counselling and health education regarding contraception Contraceptive commodity availability
Societal/public policy	Parental consent needed in accessing services Illegality of abortion services^a^	Legal access to services for emancipated minors^b^

a According to the current Kenyan constitution, abortion is illegal in all circumstances outside of emergency situations to protect the health of the mother. Even with these laws, an estimated 465,000 Kenyan women experienced abortions in 2012, and of those, 120,000 women experienced complications from the unsafe services [31].

b Currently, the Kenyan government requires parental consent for adolescents to receive reproductive health services. Many clinics provide more reproductive health services than is indicated by the current law, and in 2014 the Reproductive Health Care Bill was introduced to the Kenyan Senate that would make family planning and contraceptive services available to all adolescents, starting at 10 years of age [32,33].

### Interpersonal barriers

The providers indicated that many adolescent females fear overall community stigma of being sexually active at a young age with being seen attending the health facilities for contraceptive services, invariably implying their engagement in premarital sex. Specifically, providers expressed that the adolescents fear being labelled as sexually promiscuous by community members, including parents, peers and the providers themselves. Direct consequences of these perceived community norms on the adolescents’ health outcomes include unintended pregnancies or sexually transmitted infections (STIs).

There is fear of coming to the clinic because they are young, that coming to ask about family planning is not right for them. So they go away and become pregnant later. (Female nursing officer, sub-county hospital)

#### Stigma of sexual promiscuity in accessing contraception without a partner

Providers noted that accessing services without their sexual partner makes it difficult for adolescent females to seek contraceptive services. The providers felt that as single, unmarried females, adolescents do not feel comfortable admitting that they are sexually active. In addition, providers view these adolescents as not being serious about their relationships or having multiple concurrent relationships if they do not come to the facilities with their partner. Thus, providers felt that accessing contraceptive services is generally more challenging for the adolescent females who present to the facility without a partner.

The challenge is … they still have no partner. They are not like a couple. So for them to freely come and say that, “Me, I'm practicing sex,” is still an issue. (Male nursing officer, health center)

Providers insinuated that many male partners were fearful or unwilling to seek contraceptive services with their partners, particularly older male partners. Asking their partners to accompany them to the clinic is challenging, and adolescents are less likely to attend the health facility alone due to the stigma illustrated above. Yet, providers frequently indicated that adolescent females were more likely to seek contraception when accompanied by their partners, and attending clinic appointments together could facilitate a more open discussion about contraception between the adolescent female and her partner.

Because at times, these young girls are being exploited by the older men. You know, it's difficult for this young girl to go and tell this man, “You know, I want to go to the clinic with you,” and… even [with] the young boys [from] school – they can't come together. (Female nursing officer, dispensary)

#### Concerns of negative parental attitudes towards adolescent sexual activity

Providers also perceived adolescent females as afraid of seeking contraceptive services because of concerns of negative parental attitudes. Many adolescent females seek family planning services while obtaining their HIV care or prenatal care, and some adolescents seek health care with an older caregiver or parent. Providers felt that adolescents hesitate requesting contraceptive services as this would require disclosing their sexual activity to their parents. The adolescents worry about their parents’ reactions, most commonly accusations of promiscuity, to their request for contraception.

Some of [the adolescents] come accompanied by parents… So when they are in the clinic, instead of a child coming out openly to say, “Maybe I could be given a family planning method,” there is the mother or the father [next to them]. So it's hard for them to mention family planning. (Female clinical officer, sub-county hospital)

Because they are not yet legally married, they fear their parents. They fear being seen, and they have a feeling that, “Maybe people may know that now I'm engaging in early and unsafe sex.” (Female clinical officer, county hospital)

Although parental consent can be a considerable barrier to accessing services, health providers also reported that occasionally having parents present at the health clinic could lead to higher uptake of contraceptive services among adolescents. Some parents are aware of the consequences of unintended pregnancies and encourage their daughters to use contraception.

#### Discouragement from seeking contraceptive services due to being different from peers

The providers identified that peer attitudes towards contraception as also having a significant impact on adolescent females’ views and comfort in seeking services from the health facility. The providers felt that peer attitudes can be particularly harmful given the many stereotypes and myths held by adolescents regarding contraception. In addition, providers indicated that students who return to school with medications for HIV treatment or contraception may be viewed as “different” and stigmatized for their medication.

And also from their colleagues at school. You know, you get someone to put the implant, and… they get that stigma… “Why are you using the family planning?” such like that. And for the HIV-positive adolescents, it's a challenge because when they go back, [they get asked] “Why are you taking that medication?” (Female nursing officer, dispensary)

Although some peers discourage use of contraception, some providers found that peers brought their friends to the health facility, acting as peer leaders and advocates for other adolescents to seek contraceptive services. One provider noted, “You know, sometimes they come as a group, and that makes it easier. They fear coming as one, so they need the company” (Female nursing officer, dispensary).

#### Provider interactions and bias of adolescent sexual activity influence contraceptive services offered

These providers were also aware that provider attitudes play an important role in adolescents feeling comfortable seeking contraceptive services. They stated that creating a welcoming and non-judgmental atmosphere for adolescents increases adolescent engagement about contraception. Interestingly, providers’ perceptions of the adverse effects of contraception heavily biased which methods they recommended to adolescents. For example, several providers cited promoting abstinence or condom use over hormonal methods, such as injectable or oral contraceptives, because of their fear that adolescents will experience adverse effects from using hormonal contraceptives at a young age. They also worried that providing hormonal contraception methods would deter the adolescent females from using condoms during intercourse. Furthermore, some providers were more likely to encourage adolescents to speak with their parents before obtaining contraceptive services from the health centre, due to their bias that adolescents are too young to engage in sexual activity, evidenced by some providers who spoke of these adolescents as “children.”

The providers normally don't want to expose those who haven't given birth to family planning methods, as they fear primary infertility. We talk about condoms and abstaining more because they haven't given birth yet, and we worry about the young girls presenting with dysmenorrhea from the implants or depo (depomedroxyprogesterone acetate hormonal injection). (Male clinical officer, sub-county hospital)

It's a challenge when … you've provided the implant, and this young girl is just going to expose herself to sexual intercourse because she might not even think of using a condom … maybe these young children are going to get reinfection, or sexually transmitted diseases. (Female nursing officer, dispensary)

In addition, adolescent females’ shyness in discussing contraception with providers decreases the adolescents’ ability to ask for contraception when at the health facility. The providers noticed that adolescents are more comfortable discussing contraception following assurances of confidentiality.

When they come, I'm like their mother. So they get ashamed talking to me about issues. But if they get young [providers] almost of their age, it makes it easier for them. (Female nursing officer, sub-county hospital)

### Institutional facilitators

The providers identified several facility-level factors, such as adequate space and time to discuss contraception, free contraceptives services and appropriate contraception counselling (not limited to abstinence-only education), also help this group access contraception more readily. As one provider noted, “The moment they arrive here, we have a friendly language, we receive them with a positive attitude … we educate them, and give them the services for free” (Female nursing officer, sub-county hospital). Of the institutional facilitators, providers focused on two factors, providing youth-friendly services and integrating HIV and contraceptive services. These factors allow adolescent females living with HIV to access the health facility without the worry of meeting adults who may judge them for engaging in premarital sex and also to be able to report another reason for why they need to seek services from a health facility.

#### Targeted youth-friendly services encourage adolescents to seek contraceptive services

Many health centres are adopting youth-friendly services, from using adolescent tracking forms to having youth-specific days and services. An adolescent tracking form for adolescents living with HIV prompts providers to ask about contraception use, facilitating communication of an otherwise difficult topic for adolescents.

For the HIV-positive teenagers, we have a checklist. We are able to check about the family planning. If the child is not on family planning, we try to advise them on their options. (Male nursing officer, dispensary)

For adolescents wishing to seek contraceptive services discreetly, they come alone or at the end of the day to prevent being seen by adults in their community. Thus, adolescent clinic days can allow adolescent females living with HIV to more freely seek services without worrying about the others attending the health facility. In addition, some providers reported that helping adolescents get services rapidly so they would not be discouraged by the long wait time. These youth-friendly services create a more comfortable environment for adolescents to seek contraception.

They come usually [when] we have children or adolescent day, which is Wednesday… or they come in at odd hours, when we are almost closing, when we are almost finishing our work around 5 PM. (Male clinical officer, health centre)

#### Ease of accessing contraception through integration of HIV and contraceptive services

Providers noted that institutional integration of contraception with HIV services allowed for easier access to contraceptive services among adolescent females by removing the stigma of coming to the clinic solely for contraception. Providers indicated that many adolescents are fearful of other community members asking them why they are seeking health services, so having additional reasons to go to the health facility reduces their need to report contraceptive use to others. Providers use the adolescent's appointment for HIV care to provide additional services such as contraception, which decreases the number of trips the adolescent must make to the health facility. Integration of services has also allowed providers to reinforce counselling messages such as prevention of horizontal and vertical HIV transmission.

Like if the young people who have contracted HIV, it is good for them to take the family planning so that they can avoid unwanted pregnancies and reduce the risk of children being infected with HIV. (Female nursing officer, sub-county hospital)

But where we [capture] them is when they come for voluntary counselling and testing with their boyfriends. That is the time we trap them and talk about [contraception]. (Female nursing officer, sub-county hospital)

## Discussion

According to the providers in western Kenya, negative community norms of premarital sex were the most important barrier encountered by adolescent females living with HIV in accessing contraceptive services. Conversely, institutional factors, such as HIV and contraception service integration and youth-friendly services, were most helpful in facilitating contraceptive access.

In identifying these barriers and facilitators, healthcare providers acted as a key source of information for understanding challenges of contraceptive access among adolescents. In addition to the barriers and facilitators noted above, they also highlighted challenges such as individual knowledge, attitudes and practices; institutional clinic factors including lack of space and time; availability of health education and counselling; and free contraceptive services, which have been noted elsewhere [6,13–18]. Our study highlights findings of community norms of premarital sex, male involvement and institutional factors playing a role in the ability of adolescent females living with HIV to access contraceptive services.

Stigma about sexual promiscuity among adolescent females living with HIV was a key barrier emphasized in our study. Stigma of premarital sex, perpetuated by parents, providers and other community members has been shown to be the primary barrier in HIV-uninfected adolescents in seeking reproductive health services in resource-limited countries [5]. Our study has gone further to indicate that the fear of stigma related to engaging in premarital sex and promiscuity is multifactorial, coming from a variety of different individuals in these adolescents’ social network. This further compounds the stigma these adolescents already face due to their HIV-positive status and the possibility that their HIV infection was obtained through sexual transmission [1,34]. The fear of the stigma is based on perceptions of community norms about when adolescents should be engaging in sexual activity. School-based interventions in resource-limited settings have been helpful in increasing HIV knowledge, changing norms among peers and leading to behaviour change, such as increased rates of condom use [7,35,36]. Indeed, some providers in our study noted that they were already going to schools to increase adolescent females’ knowledge about contraception. However, these school-based interventions do not reach parents and community members. Thus, it is important for HIV programmes to engage not only with adolescent females and their peers in schools, but also with parents and community members to increase contraception uptake.

The challenge of receiving contraceptive services with partners was emphasized by the health providers in our study, and there is considerable literature on the challenges and potential benefits of male involvement in contraception care. Although many women face challenges involving their male partners, involving partners is particularly difficult for adolescent females living with HIV, who often lack control in their relationships, particularly with older male partners [6]. However, coming to the health facility for contraceptive services without a male partner increased the perceived stigma of sexual promiscuity among these adolescent females, and thus male involvement may be instrumental in increasing contraception uptake. Most men want to be involved in contraception decision-making, and creating a welcoming environment for men can help them feel invested in contraceptive use [37].

Providers noted that youth-friendly services enable adolescent females living with HIV to seek appropriate contraceptive care. Many adolescents, including HIV-uninfected adolescents, receive health services either through paediatric or adult services, which are not tailored to their specific needs as adolescents [4]. However, youth-friendly services lead to improved knowledge and increased contraception use among adolescents [4,38].

A specific element of youth-friendly services is training providers to understand adolescent psychological and social development, adolescent sexuality and non-biased communication strategies, which can increase providers’ competency in providing youth-friendly services [39]. Providers in Kenya serving HIV-uninfected adolescents acknowledged that improved understanding of adolescents’ reproductive health needs would help encourage adolescents to seek services, and such themes are likely just as relevant to adolescents living with HIV [18]. Indeed, supportive providers appear key in helping adolescent females living with HIV prevent STIs, unintended pregnancies and vertical transmission of HIV [40]. Providers in our study illustrated some of their own biases, particularly regarding provision of hormonal contraception methods to adolescent females due to potential future infertility; staff training to address such misconceptions could help providers counsel adolescents appropriately for more effective contraception methods.

Finally, providers indicated that HIV and contraception integration is an essential part in ensuring that adolescent females living with HIV are able to access HIV and contraception care when they visit health facilities. Indeed, literature has shown that integrating these services not only increases use of contraception, but also prevents unintended pregnancies and, therefore, vertical transmission of HIV [41–44]. As adolescent females living with HIV attend health facilities for their HIV care, this group is well positioned for facility-based interventions to improve reproductive health services including the provision of effective contraception. In addition, HIV and contraception integration has been shown to strengthen male involvement in contraception [1,45,46].

Our study has several strengths, including sampling health providers from a range of different health facilities, from small, rural dispensaries to county hospitals. The open-ended questions allowed for health providers to voice different barriers and facilitators, and our sample size demonstrated adequate saturation in the major themes identified. Nonetheless, our study has a few limitations. This study interviewed providers as opposed to the female adolescents living with HIV themselves, so our data are based on providers’ perceptions and biases of adolescent sexuality. However, studies investigating adolescents’ perspectives have identified similar barriers and facilitators as our study, illustrating providers are a reliable source for assessing barriers and facilitators to care among adolescents [6,10,13–18]. Furthermore, providers are likely more aware of facility-level factors influencing contraception uptake than adolescents, which allows them to uniquely comment on the impact of such factors on contraceptive use.

## Conclusions

Our study identifies social norms of promiscuity, male involvement and institutional factors as the major barriers and facilitators of contraception use among adolescents living with HIV. Given the high prevalence of HIV among adolescents in western Kenya and their substantial risk of unintended pregnancy, it is imperative that adolescents’ access to contraceptive services be optimized. Understanding contraceptive use among adolescent females living with HIV is not only key in decreasing their rates of unintended pregnancy and vertical transmission of HIV, but may be linked with enhanced educational and economic opportunities [7,9,11]. Health facilities should continue to provide appropriate counselling and contraceptive services for adolescent females in a youth-friendly manner and integrate HIV and contraception services to encourage more adolescents living with HIV to obtain contraception while receiving HIV care. As importantly, HIV programmes need to recognize that relationships between adolescent females living with HIV and their parents, partners, health providers and communities are crucial in enhancing adolescents’ use of contraception, and changing social norms regarding premarital sex is a difficult but critical element of increasing contraception use among this group.

## Supplementary Material

Barriers and facilitators adolescent females living with HIV face in accessing contraceptive services: a qualitative assessment of providers’ perceptions in western Kenya

Barriers and facilitators adolescent females living with HIV face in accessing contraceptive services: a qualitative assessment of providers’ perceptions in western Kenya
