# Supplementary material for: Barriers and facilitators adolescent females living with HIV face in accessing contraceptive services: a qualitative assessment of providers’ perceptions in western Kenya
Source: J Int AIDS Soc. 2015 Sep 18;18(1):20123. doi: 10.7448/IAS.18.1.20123 (PMC4575411; doi:10.7448/IAS.18.1.20123)
Supplement: Barriers and facilitators adolescent females living with HIV face in accessing contraceptive services: a qualitative assessment of providers’ perceptions in western Kenya [file JIAS-18-20123-s001.pdf]

Supplemental File 2 – Provider Questionnaire

FACES Family Planning Facility-Level Questionnaire

Interviewer, please read the script below to your interviewee prior to starting the questionnaire:

“The below questionnaire is meant to understand if Family Planning (FP) services are offered at this HIV care facility. By “FP services,” we mean methods of contraception to prevent pregnancy. FP services may include actual provision of contraceptive methods or counseling about these types of methods. Such methods may include condoms, pills, depo/injections, IUCDs, implants, or surgical procedures such as tubal ligation or vasectomy.

The first part of the questionnaire is meant to help us understand what types of FP services you have. The second part of the questionnaire will ask your opinions on specific topics related to FP.

Your responses to the below questions will be kept anonymous and confidential. Do you have any questions? Let’s begin.”

1. Today’s Date:

2. Facility Name:

3. District or Subdistrict Location:

4. Number of persons aiding in completion of questionnaire:

5. Interviewee title(s) or role(s): Check all who aided in completion of this questionnaire.

___a. Medical officer (MO)

___b. Facility officer (CO)

___c. Nurse officer (general, NO)

___d. FP-specific nurse officer

___e. Pharmacist

___f. Facility-in-charge or Facility administrator

___g. Other, specify:

PART I: FP services at HIV care facility

Interviewers, please read: "'FP needs' are defined as a need for family planning or specifically deciding when to have children and when to delay having children."

A. Screening for FP needs

6. Are HIV positive **women** who are attending this HIV care facility screened for FP needs?

___a. No

___b. Yes (Integration Point: 1)

7. Are HIV positive **men** who are attending this HIV care facility screened for FP needs?

___a. No

___b. Yes

8. Are **couples** screened for FP needs together?

___a. No

___b. Yes

9. In general, **who** does FP needs screening at your HIV care facility?

10. In general, **when** does FP needs screening occur for the client?

___a. at check-in or registration for the HIV care facility

___b. in the waiting area while waiting to see a provider

___c. with the provider (e.g. nurse, CO, or MO)

___d. by the pharmacist when dispensing medications

___e. Other, specify:____

11. In general, **where** does FP needs screening physically occur?

___a. at the registration counter

___b. in the waiting area

___c. in a room with a provider

___d. at the pharmacy

___e. Other, specify:____

12. What types of questions are asked when screening patients for FP needs?

13. If the FP needs screen is positive, e.g. a patient says he or she is interested in delaying pregnancy, what is done next?

B. Education and Counseling of FP

14. Does your facility provide any education or counseling regarding FP methods?

___a. No

___b. Yes (Integration Point: 1)

15. Are couples counseled **together** regarding FP methods?

___a. No

___b. Yes (Integration Point: 2)

16. In general, **how** are clients educated or counseled about FP methods?

17. In general, **who** does the education or counseling about FP methods?

18. If couples have different fertility desires (e.g. one partner is interested in delaying pregnancy while the other is not), what is done next?

C. Provision of FP

19. Are any FP commodities (including condoms) provided at this HIV care facility?

___a. No

___b. Yes

If yes, go to #19a. If no, skip to #31. Please note that all the facilities should be providing at least condoms.

19a. If yes, which of the following are provided at this HIV care facility? Check all that apply: (Integration Point: 1, if any excluding condoms)

___a. Male condoms

___b. Female condoms

___c. Birth control pills or oral contraceptive pills

___d. IUD or IUCD

___e. Implants

___f. Injectables or depo

___g. Emergency contraception

___h. Other, specify:

20. Are FP surgical services such as tubal ligation, hysterectomy or vasectomy provided at this HIV care facility by your staff?

___a. No

___b. Yes

20a. If yes, which ones: Check all that apply.

___a. Hysterectomy

___b. Bilateral tubal ligation (BTL)

___c. Vasectomy

___d. Other, specify:

20b. If no, then do other groups or organizations come to your facility to provide these services, such as Marie Stoopes?

___a. No

___b. Yes

20c. If yes, which ones: Check all that apply.

___a. Hysterectomy

___b. Bilateral tubal ligation (BTL)

___c. Vasectomy

___d. Other, specify:

21. Which FP commodities are in-stock and available **today**? Check all that apply. (Integration Point: 1, if any excluding condoms)

___a. Male condoms

___b. Female condoms

___c. Birth control pills or oral contraceptive pills

___d. IUD or IUCD

___e. Implants

___f. Injectables or depo

___g. Emergency contraception

___h. Other, specify:

22. Are FP services provided by the **same provider** also providing HIV care services? (Integration Point: 2)

___a. No

___b. Yes

23. Are FP services provided in the **same room** as the provision of HIV care services? (Integration Point: 2)

___a. No, and why not?____

___b. Yes

24. Who generally provides FP services at your facility? Check all that apply.

___a. MO

___b. CO

___c. Nurse

___d. Pharmacist or Pharm tech

___e. Other, specify:

25. Please indicate the total number of providers working at this HIV care facility: _____

26. Indicate the number of providers working at this HIV care facility who have been **trained for** and can provide the following:

a. Pills: _____

b. Depo: _____

d. IUCDs: _____

e. Implants: _____

f. Surgical procedures such as vasectomy and tubal ligation: _____

27. Indicate the number of providers working **today** who have been trained for and can provide the following:

a. Pills: _____

b. Depo: _____

d. IUCDs: _____

e. Implants: _____

f. Surgical procedures such as vasectomy and tubal ligation: _____

28. Of the days of the week when HIV care services are offered, how many of those days are FP services also offered? ____days out of ____total days

29. Of those days, are FP services offered part of the day or all day?

___a. Part of the day

___b. All of the day

30. Do the days of FP services provision coincide with HIV services provision?

___a. No, they are always offered at separate times

___b. Sometimes, they are offered at the same time but not always (Integration Point: 1)

___c. Always, FP services are always offered at the same time as the HIV services provision (Integration Point: 2)

D. Referral for FP

31. Do you refer your patients to other facilities for FP commodities or services that your facility does not provide?

___a. No

___b. Yes

31a. If yes, where do you refer these patients and for what reasons? Check all that apply.

___a. District hospital; list specific reasons for referral: ________________

___b. Maternal and child health (MCH) facility; list specific reasons for referral: ____

___c. Another HIV facility; list specific reasons for referral: ________________

___d. Other, specify: ______________; list specific reasons for referral: ____

31b. Are any of the facilities where you refer these patients located on the same grounds as your HIV care facility? (Such as the MCH facility?)

___a. No

___b. Yes, and which ones?____

32. Do patients have to pay for any FP commodities at your facility? If so, approximately how much?

Mark yes or no for each method, and indicate approximate cost.

a. Male condoms Y/N; Cost: ____

b. Female condoms Y/N; Cost: ____

c. Birth control pills or oral contraceptive pills Y/N; Cost:__________________________

d. IUCD Y/N; Cost: ____

e. Implants Y/N; Cost: ____

f. Injectables or depo Y/N; Cost: ____

g. Emergency contraception Y/N; Cost: _______________________________________

33. Any other information you would like to provide before we move on to the next section about your opinions on FP provision?

PART II: Provider attitudes and opinions

Interviewers, please read: "In this next section, I will ask you about your opinions and thoughts on barriers and facilitators for FP at your facility."

A. Barriers or facilitators of FP (general)

34. What are **barriers** to HIV positive individuals obtaining FP services at your HIV care facility?

35. What are factors that **facilitate** HIV positive individuals obtaining FP services at your HIV care facility?

36. What are the **challenges** in providing FP services at your HIV care facility?

37. How can organizations like FACES help you overcome those challenges?

38. Have you experienced any commodity stock-outs in the last month?

___a. No

___b. Yes

38a. If yes, list commodities that were out of stock:

39. Which stock management tools does your facility currently use (list all that apply):

B. Barriers or facilitators of FP for men and male involvement in FP

40. Are there specific **barriers** for HIV positive men in being **screened** for FP needs at your HIV care facility? With their female partners?

41. Are there specific **barriers** for HIV positive men in **obtaining** FP commodities at your HIV care facility? With their female partners?

42. Are there specific factors that **facilitate** HIV positive men in being **screened** for FP needs at your HIV care facility? With their female partners?

43. Are there specific factors that **facilitate** HIV positive men in **obtaining** FP commodities at your HIV care facility? With their female partners?

44. What are the **challenges** of providing FP services to HIV positive men and their partners at your HIV care facility?

45. Does your HIV care facility do anything in particular to encourage men to **think about** FP with their partners?

46. In your opinion, are couples more likely to utilize FP commodities if they are screened or counseled together for FP? Why or why not?

C. Barriers or facilitators of FP for girls and young women

47. At what age generally do girls and young women (females less than age 20) become sexually active in the community that your facility serves?

48. Do girls and young women (females less than age 20) have FP needs that are different from women who are older in the community that your facility serves? Why or why not?

49. Are there specific **barriers** for HIV positive girls and young women (females less than age 20) in being **screened** for FP needs at your HIV care facility?

50. Are there specific **barriers** for HIV positive girls and young women (females less than age 20) in **obtaining** FP commodities at your HIV care facility?

51. Are there specific factors that **facilitate** HIV positive girls and young women (females less than age 20) in being **screened** for FP needs at your HIV care facility?

52. Are there specific factors that **facilitate** HIV positive girls and young women (females less than age 20) in **obtaining** FP commodities at your HIV care facility?

53. What are the **challenges** of providing FP services to HIV positive girls and young women (females less than age 20) at your HIV care facility?

54. Does your HIV care facility do anything in particular to encourage girls and young women (females less than age 20) to **think about** FP?

55. In your opinion, would girls and young women (females less than age 20) who are sexually active bring in their male partners to be screened or counseled together for FP? Why or why not?

D. FP commodities provision at community-level

56. Do you think it is appropriate to provide FP commodities at the community-level, for example, during community-based health campaigns? At homes? Why or why not?

57. Do you think members of your community would feel comfortable obtaining family planning counseling or commodities at community-based health campaigns? At homes? Why or why not?

58. Do you think **men** in particular would feel comfortable obtaining family planning counseling or commodities at community-based health campaigns? At homes? Why or why not?

59. Do you think men would be more or less comfortable obtaining FP counseling or commodities at a health campaign or at home compared to at your HIV care facility? Why?

60. Would you feel comfortable **counseling** individuals or couples regarding family planning at community-based health fairs or campaigns? Why or why not?

61. Would you feel comfortable **providing** FP commodities at community-based health fairs or campaigns? Why or why not?

62. Would you feel comfortable providing the following FP methods at community-based health fairs or campaigns? Circle yes or no. Why or why not? Indicate reasons by each response.

a. Male condoms Y/N; Reason: ____

b. Female condoms Y/N; Reason: ____

c. Birth control pills or oral contraceptive pills Y/N; Reason: ______________________

d. IUD or IUCD Y/N; Reason:
____

e. Implants Y/N; Reason: ____

f. Injectables or depo Y/N; Reason: ____

g. Emergency contraception Y/N; Reason: __________________________________

h. Referrals for surgical procedures Y/N; Reason: _____________________________

63. Is there anything else you would like to share before we finish?

Interviewers, please read: "Thank you for your time and help in completing this questionnaire."

64. Level of FP/HIV Integration Scale (Circle each point for the item applicable; please only select either 1 or 2 points for items 2 and 7)

**Item**	**Point**
1. HIV positive women screened for FP needs	1
2. Education or counseling regarding FP methods	
FP methods counseling only provided to individuals	1
FP methods counseling provided to couples together	2
3. Provide at least one form of modern contraception (excluding condoms)	1
4. FP commodities in-stock the day of administering this questionnaire	1
5. FP services provided by the same provider/provider as HIV care services	2
6. FP services provided in the same room as the provision of HIV care services	2
7. FP service provision days coincide with HIV care provision days:	
Sometimes	1
Always	2
Fill in Total Score (maximum score 11)	___

65. FP integration by CCC Facility Category (Circle Yes or No for each category)

Category 1	Promotes FP services, Offers for FP screening, Combined oral contraceptives, Emergency oral contraception within the HIV clinic	Yes/No
Category 2	All category 1 plus injectable contraceptives	Yes/No
Category 3	All Category 1, 2 plus IUCD, Implant	Yes/No
Category 4	All category 1,2,3 plus surgical contraception and follow-up	Yes/No
