# Supplementary material for: Barriers and facilitators adolescent females living with HIV face in accessing contraceptive services: a qualitative assessment of providers’ perceptions in western Kenya
Source: J Int AIDS Soc. 2015 Sep 18;18(1):20123. doi: 10.7448/IAS.18.1.20123 (PMC4575411; doi:10.7448/IAS.18.1.20123)
Supplement: Barriers and facilitators adolescent females living with HIV face in accessing contraceptive services: a qualitative assessment of providers’ perceptions in western Kenya [file JIAS-18-20123-s002.pdf]

Supplemental File 1 – Facility Information

HIV Integration Provided by FACES

FACES provides technical support to over 130 health facilities in western Kenya, spanning Homabay, Kisumu, and Migori counties, to allow for HIV service integration into maternal and child health programs. Over 121,000 patients have enrolled in care and over 51,000 have initiated antiretroviral treatment [23]. As detailed in Odeny et al.'s description of the FACES program, specific cornerstones of HIV integration championed by FACES include:

- "Clinic Space and Clinicians: Before integration, HIV-positive patients were seen separately on one to four dedicated days each week. After integration, they were seen on any day of the week in the general outpatient clinic; all patients followed a uniform patient flow, and patient load was evenly spread out among the existing staff.
- Health Education: Integrated health education was conducted for all patients regardless of HIV status in a single shared waiting bay. This approach was seen as a way of reducing stigma against HIV and broadening health education reach.
- Pharmacy/Supplies: With integration, all drugs were stored in the same storage space and dispensed from the same pharmacy; supply and inventory management at the clinics under evaluation were also integrated. The integrated pharmacy became the central repository and dispensing area for therapeutic food supplements (for eligible HIV-infected patients and HIV-negative children of certain ages) that would otherwise have to be stored in a separate food store. Orders for all drugs and food supplements were made within the same supply-chain network. Training and mentorship for pharmacy and supplies management were offered to both Ministry of Health (MOH) and FACES staff who then worked together in the integrated pharmacy and were able to share the daily workload.
- Laboratory: Introduction of HIV services brought with it a well-equipped central laboratory at the district hospital; after integration, these lab facilities were used to conduct other tests not necessarily related to HIV. For example, a new automated biochemistry machine to be used for measuring liver enzymes to monitor the

progress of patients on ART was also used for other patients when clinically indicated. Supportive supervision for the district lab network prior to integration was infrequently conducted by the MOH District Medical Laboratory Technician; however, after integration it was conducted by a combined FACES and MOH team. Laboratory supervision was more frequent and regular after integration because FACES had a more reliable transport system.

- Clinical Mentorship and Training: Before integration, only providers working in the HIV clinic underwent mentorship by experienced providers from FACES during weekly visits. After integration, all providers underwent mentorship. All clinical officers and nurses were offered training for HIV-related and non-HIV-related topics such as Rational Use of ART in Adults, Comprehensive Pediatric HIV Care, Integrated Management of Adult and Adolescent Illness, Prevention of Mother to Child Transmission of HIV, and Psychosocial Counseling for Pediatrics." [24]

Health care is provided at county hospitals, sub-county hospitals, health centers, and dispensaries. An overview of the services provided at each facility type based on the Kenya Service Provision Assessment in 2010 is provided below:

> "**Health sub-district**
>
> District and sub-district, or primary, hospitals offer referrals and guidance to the health centres. At the same time, they concentrate on the core functions required of their level, which include curative and rehabilitative activities.
>
> **Health centres**
>
> Health centres focus their attention on preventive and curative services, mostly adapted to local needs. They also offer ambulatory services to the communities. Health centres have on staff registered clinical officers, registered community health nurses, laboratory technicians, and pharmaceutical technologists.
>
> **Dispensaries**
>
> Dispensaries are meant to be the first line of contact with the community. This function is shared by the health centres. The dispensaries provide a broad spectrum of preventive health services, which is critical to achieving the health

sector reform focus on individual life-style and the community. They also offer basic curative services. Dispensaries are staffed with enrolled community nurses and community health extension workers." [25]

Of the 130 FACES-supported facilities, Table 3 lists the 21 FACES-supported health facilities included in the provider questionnaire.

Table 3. 21 FACES-supported Health Facilities included in Provider Questionnaire.

County	Facility Site	Facility Type
Kisumu	Nyahera	Sub-County Hospital
	Rabour	Health Center
	Lumumba	Health Center
	Tuungane	Health Center
	Nyalunya	Dispensary
Migori	Migori District Hospital	County Hospital
	Rongo District Hospital	Sub-County Hospital
	Macalder	Sub-County Hospital
	Karungu	Sub-County Hospital
	Ongo	Health Center
	Oyani	Health Center
	Muhuru	Health Center
	Bware	Dispensary
	Kolwal	Dispensary
	Saro	Dispensary
	Agenga	Dispensary
Homabay	Sindo District Hospital	County Hospital
	Mbita District Hospital	Sub-County Hospital
	Sena	Health Center
	NYS	Dispensary
	Obalwanda	Dispensary

Specific Contraceptive Services Provided by FACES-supported Health Facilities

Details regarding the specific contraceptive services provided by the 21 FACES-supported health facilities included in study sample are reported below based on provider reports from the first portion of the questionnaire, which had structured questions regarding contraceptive screening, education and counseling, commodities, provider workforce, and integration of services (Table 4). The provider-reported data indicated that almost all of the facilities provided screening, education, and counseling to all women living with HIV, as well as their partners. Facilities offered on average 6 different contraception options, although stock outs were more likely in the health centers and dispensaries compared with the county and sub-county hospitals (71.4% and 57.1%

versus 47.1%). Additionally, county and sub-county hospitals were more likely to have more providers on staff to provide health services, although a small proportion of providers were trained in IUD placement across all levels of health facilities. Finally, health centers and dispensaries were more likely to offer HIV and contraceptive services in the same room by the same provider, likely due to the restrictions in number of providers and space available at these locations in comparison to the larger county and sub-county hospitals.

Table 4. Contraceptive Services provided by 21 FACES-supported Health Facilities.

Service Provision	County and Sub-County Hospitals (N or %)	Health Centers (N or %)	Dispensaries (N or %)
Screening for Contraceptive Needs			
HIV Positive Women Screened	100%	100%	100%
HIV Positive Men Screened	83.3%	78.6%	85.7%
Couples Screened Together	94.4%	92.9%	100%
Education and Counseling on Contraception			
HIV Positive Women Educated	100%	100%	100%
Couples Educated Together	94.4%	92.9%	100%
Contraceptive Commodities			
Male Condoms	100%	100%	100%
Female Condoms	83.3%	64.3%	42.9%
Oral Contraceptive Pills	100%	100%	100%
IUD/IUCDs	100%	92.9%	85.7%
Implants	100%	100%	100%
Injectables (Depo Provera)	100%	100%	100%
Emergency Contraception	83.3%	57.1%	71.4%
Surgical Services (tubal ligation, hysterectomy, vasectomy)	38.9%	0%	0%
Contraceptive Cost			
No Cost	100%	100%	100%
Stock of Contraceptives			
Mean Number of contraceptive choices in stock on day of interview	6.1	5.9	5.7
Percentage with Contraceptive stock-outs within last month	47.1%	57.1%	71.4%
Provider Work Force			
Mean Number of Total Providers	10.4	6.9	3.3
Mean Number of Providers trained to offer oral contraceptive pills	10.1	6.7	3.1
Mean Number of Providers trained to offer IUD/IUCDs	6.9	3.8	1.6

Mean Number of Providers trained to offer implants	9.2	5.2	3.1
Mean Number of Providers trained to offer injectables	10.0	6.3	2.9
Mean Number of Providers trained to offer surgical services	0.7	0.0	0.0
Availability of Services			
Mean Number of days per week contraceptive services offered	4.8	4.9	4.9
Referral for Services			
Referral for other contraceptive services	83.3%	92.9%	85.7%
Integration of HIV and Contraceptive Services			
HIV and contraception services offered by the same provider	77.8%	100%	100%
HIV and contraception services offered in the same room	50.0%	71.4%	100%
HIV and contraception services offered on the same day	94.4%	92.9%	85.7%
